# Interfacial Effect on Photo-Modulated Magnetic Properties of Core/Shell-Structured NiFe/NiFe_2_O_4_ Nanoparticles

**DOI:** 10.3390/ma15041347

**Published:** 2022-02-11

**Authors:** Wenda Zhou, Mingyue Chen, He Huang, Guyue Wang, Xingfang Luo, Cailei Yuan, Jingyan Zhang, Yanfei Wu, Xinqi Zheng, Jianxin Shen, Shouguo Wang, Baogen Shen

**Affiliations:** 1 School of Materials Science and Engineering, Beijing Advanced Innovation Center for Materials Genome Engineering, University of Science and Technology Beijing, Beijing 100083, China; wdzhou@xs.ustb.edu.cn (W.Z.); mychen@xs.ustb.edu.cn (M.C.); hhuang@ustb.edu.cn (H.H.); ywang@xs.ustb.edu.cn (G.W.); jyzhang@ustb.edu.cn (J.Z.); yanfeiwu@ustb.edu.cn (Y.W.); zhengxq@ustb.edu.cn (X.Z.); jxshen@ustb.edu.cn (J.S.); 2 Jiangxi Key Laboratory of Nanomaterials and Sensors, School of Physics, Communication and Electronics, Jiangxi Normal University, Nanchang 330022, China; xfluo@jxnu.edu.cn; 3 Beijing National Laboratory for Condensed Matter Physics, Institute of Physics, Chinese Academy of Sciences & University of Chinese Academy of Sciences, Beijing 100190, China; 4 Institute of Rare Earths, Chinese Academy of Sciences, Ganzhou 341000, China

**Keywords:** bimagnetic core/shell, interfacial effect, photo-modulation

## Abstract

Photo-modulated magnetism has become an emerging method for technological applications, such as magneto-optical devices. In this work, by introducing oxygen during rapid thermal annealing, NiFe/NiFe_2_O_4_ core/shell nanoparticles were successfully fabricated by pulsed laser deposition. Obvious photo-modulated ferromagnetism was observed in core/shell nanoparticles confined in Al_2_O_3_ film. Theoretical and experimental investigations indicate much more photogenerated electrons are captured at the interface of NiFe/NiFe_2_O_4_ compared with NiFe nanoparticles due to interfacial effect, resulting in the improved ferromagnetism under light irradiation. This work provides a promising strategy for optical engineering design of optical information storage, high-speed wireless communication, and magneto-optical semiconductor devices.

## 1. Introduction

Bimagnetic core/shell nanocomposites have attracted particular attention because of their promising application prospects in diverse fields, such as magnetic devices, biomedicine, energy storage, and spin-related catalysis [1,2,3,4,5,6,7,8]. In fact, core/shell architecture has been considered an attractive approach to developing novel properties of various nanomaterials [9,10,11]. For the material design of bimagnetic nanocrystals, in addition to integrating the characteristics of the core and shell, interfacial exchange (interfacial effect) is vital for the eventual realization of designed and tunable functionalities [12,13,14]. However, a fundamental understanding of the core/shell interfacial effect remains a major challenge. On the other hand, tunable magnetism is one of the most widely studied issues in the application and development of spintronics [15,16,17,18]. It has been proposed that many strategies such as magnetic field, spin-orbit engineering, thermal gradient, chemical functionalization, strain, and electric field are greatly helpful to realize the manipulation of magnetism [15,16,17,18,19,20]. Apart from the aforementioned modulation techniques, photo-modulation of magnetism has become an emerging method for technological applications, especially in high-speed wireless communication [21,22,23,24,25]. This approach has the advantages of large storage capacity and low cost, which has attracted extensive research recently [22,23,24,25,26]. In this case, although the interfacial electronic structure of core/shell architecture inherently affects the magnetic properties of bimagnetic nanoparticles and provides an ideal platform for optical adjustment function, the photo-modulation of magnetism in bimagnetic core/shell nanoparticles remains a relatively unexplored area.

Permalloy (NiFe) is a typical soft magnetic material widely used in transformer cores and magnetic recording heads [27]. Nickel ferrite (NiFe_2_O_4_) with a high Curie temperature and tunable bandgap are applicable in magnetic refrigeration, magnetic resonance imaging, biomedical applications, and catalysis [25,28,29]. Integrating both materials to create bimagnetic core/shell nanocomposites may bring innovative properties and intriguing applications [30]. More interestingly, this bimagnetic core/shell nanocomposite may also provide a platform for photo-modulation by interfacial effect. In this work, the NiFe/NiFe_2_O_4_ core/shell nanoparticles were prepared through a pulsed laser deposition (PLD) technique and rapid thermal annealing under an oxygen atmosphere. With the interfacial effect of core-shell structure, the magnetic properties of NiFe/NiFe_2_O_4_ nanoparticles can be significantly manipulated by photo-illumination, but no obvious change in NiFe nanoparticles under photo-illumination. Density functional theory (DFT) calculation indicates that the photo-modulated effect is associated with the charge accumulations at the interface of core/shell architecture. The robust photo-modulated ferromagnetism can help accomplish high-speed and wireless nonvolatile storage and logic operations. This work demonstrates the tunability of the core/shell interfacial effect and expands the potential applications of bimagnetic nanoparticles to optical information storage and semiconductor spintronics.

## 2. Materials and Methods

NiFe/NiFe_2_O_4_ nanoparticles confined in Al_2_O_3_ films were synthesized by the PLD system (LMBE450A, SKY, Shenyang, China) and rapid thermal annealing. Briefly, the NiFe target (10 mm × 20 mm, 99.99% purity) was glued to the Al_2_O_3_ target (60 mm in diameter, 99.99% purity) by silver glue. The p-type Si substrates were cleaned with deionized water and followed by acetone. During the deposition process, the assembled NiFe/Al_2_O_3_ target rotated slowly around the central axis, and these two components were evaporated to Si substrate alternately by a laser beam. The composite target was ablated by KrF pulsed laser with a wavelength of 248 nm for 15 min after the chamber pumped down to 1.5 × 10^−7^ Torr. During the deposition, the composite target was slowly rotated. Then, the samples were annealed at 600 °C for 120 s under oxygen partial pressure of about 1 Pa. The synthesis condition of NiFe nanoparticles is the same as NiFe/NiFe_2_O_4_ nanoparticles, but without oxygen during the rapid thermal annealing process. Transmission electron microscopy (TEM) characterizations were performed by FEI Talos f200× (Thermo Fisher, Waltham, MA, USA) microscope operated at 200 kV. X-ray photoelectron spectroscopy (XPS) was examined using the XSAM800 spectrometer (Kratos, Manchester, UK) employing Al Kα radiation. The XPS binding energies were calibrated by the C 1s peak (284.8 eV). The physical property measurement system (PPMS, Ever Cool II, Quantum Design, San Diego, CA, USA) with a vibrating sample magnetometer option was used to investigate the magnetic properties of the synthesized samples.

## 3. Results and Discussion

**Figure 1:**
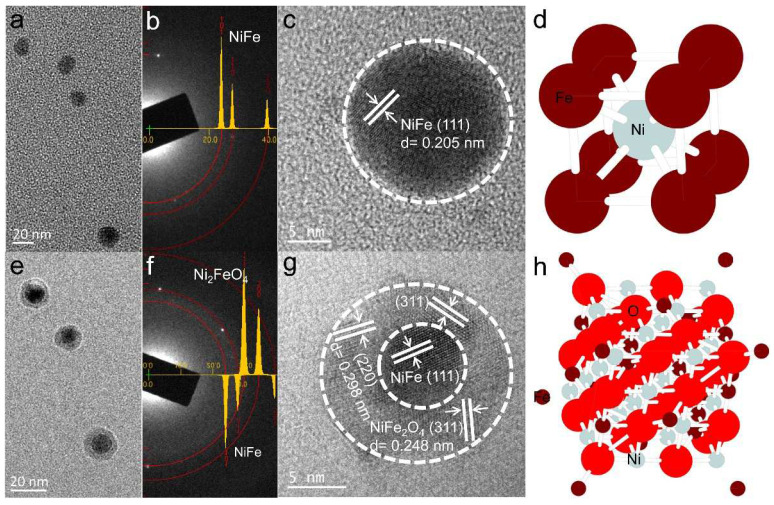
(**a**,**e**) lanar low magnitude TEM images, (**b**,**f**) electron diffraction patterns, (**c**,**g**) HRTEM images, (**d**,**h**) unit cell schematic diagrams of NiFe nanoparticles and NiFe/NiFe_2_O_4_ core/shell nanoparticles in amorphous Al_2_O_3_ matrix, respectively.

Figure 1a shows the low magnification TEM image of as-synthesized NiFe/Al_2_O_3_ nanofilm. Obviously, several NiFe nanoparticles with an average size of ~20 nm can be observed in the amorphous Al_2_O_3_ matrix. As shown in Figure 1b, the selected area electron diffraction (SAED) pattern of NiFe nanoparticles shows a good match with the simulated results obtained by TEM simulator software (JAVA Electron Microscope Simulation (JEMS)) [24]. It clearly demonstrates that the NiFe nanoparticle has a cubic structure with the p4/mmm space group (Figure 1d). The high-resolution TEM (HRTEM) image in Figure 1c further shows the successful formation of NiFe nanoparticles with a size of about 20 nm, and the inter-planar spacing (0.205 nm) corresponds to the (111) crystal plane of NiFe [31]. By introducing oxygen during rapid thermal annealing, the NiFe/NiFe_2_O_4_ core/shell nanoparticles can be successfully fabricated, as shown in Figure 1e. The average size of NiFe/NiFe_2_O_4_ core/shell nanoparticles is ~20 nm found from Figure S1 (the histogram of the NiFe/NiFe_2_O_4_ nanoparticles size distribution in Supporting Information). As shown in Figure 1f, the diffraction patterns for NiFe and NiFe_2_O_4_ match with their simulated diffraction patterns by TEM JEMS software. The experimental and simulated electron diffraction patterns confirmed that the core/shell nanoparticles consisted of cubic NiFe (space group p4/mmm) and cubic NiFe_2_O_4_ (space group Fd-3mS, Figure 1h). The HRTEM image (Figure 1g) reveals that the NiFe/NiFe_2_O_4_ core/shell structure is composed of a single crystal NiFe core and uniform polycrystalline NiFe_2_O_4_ shell with different lattice orientations [32]. It is reasonable to conclude that the oxidation only takes place on the surface of NiFe nanoparticles, rather than penetrating the internal NiFe nanoparticles during the annealing process to form NiFe_2_O_4_ shell. Besides, the formation of NiFe_2_O_4_ shell is also confirmed by Raman spectra (Figure S2 in Supporting Information) [25]. Therefore, by adjusting the oxygen atmosphere, the microstructures and morphologies of nanoparticles can be significantly influenced, and the growth of NiFe and NiFe/NiFe_2_O_4_ core/shell structure can be well controlled.

**Figure 2:**
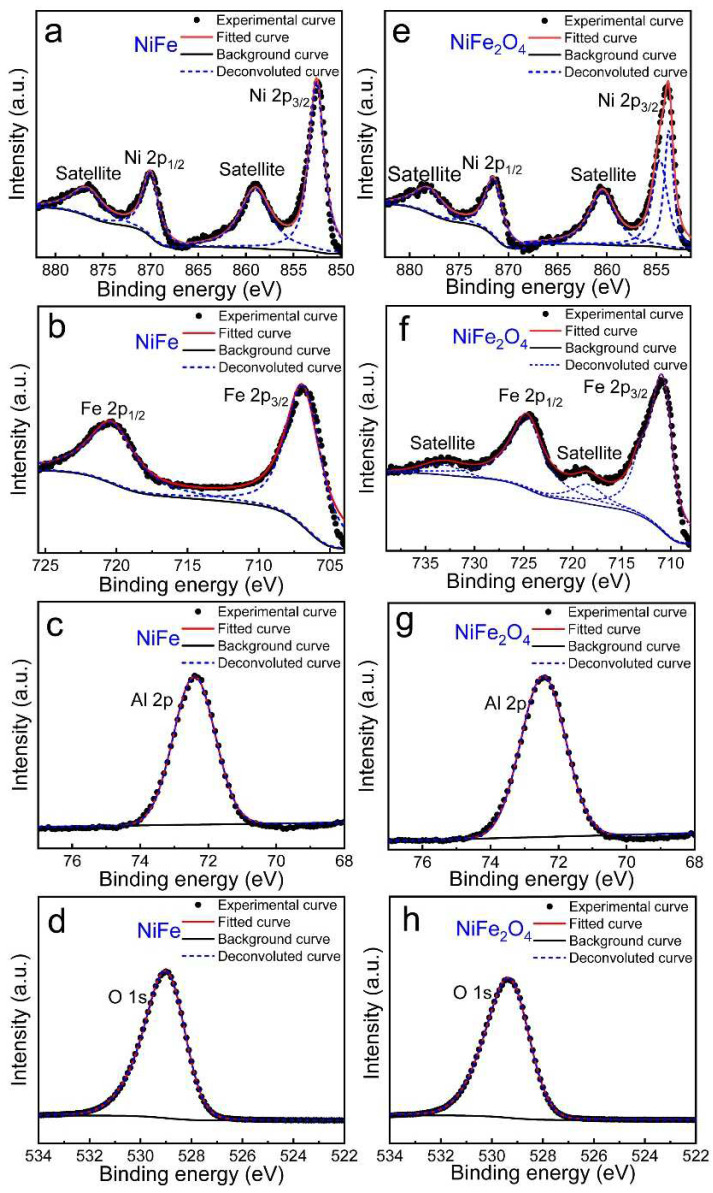
XPS spectra of (**a**,**e**) Ni 2p, (**b**,**f**) Fe 2p, (**c**,**g**) Al 2p, (**d**,**h**) O 1s of NiFe nanoparticles and NiFe/NiFe_2_O_4_ core/shell nanoparticles, respectively.

XPS technique was adopted to clarify the chemical compositions and stoichiometry of NiFe and NiFe_2_O_4_ nanoparticles in the Al_2_O_3_ matrix. As displaced in Figure 2a, for NiFe in the Al_2_O_3_ matrix, the binding energy of Ni 2p_3/2_ and Ni 2p_1/2_ core level is located at 852.6 eV and 870.1 eV, respectively, where its satellite peaks at 859.0 eV and 876.9 eV indicate a Ni^0^ valence state in NiFe [33]. Likewise, the binding energy of Fe 2p_3/2_ and Fe 2p_1/2_ locates at about 706.6 and 720.5 eV (Figure 2b), respectively, in good agreement with the Fe^0^ counterpart reported before [34]. Besides, the main peak of O 1s is centered at 529.0 eV (Figure 2c) and of Al 2p at 72.4 eV (Figure 2d) for NiFe in Al_2_O_3_ matrix implies that NiFe shows no chemical interaction with the surrounding Al_2_O_3_ matrix [24], in agreement with the TEM results. In the case of NiFe_2_O_4_ nanoparticles in the Al_2_O_3_ matrix, the XPS spectra of Ni 2p and Fe 2p were also included. As shown in Figure 2e, the main band of Ni 2p_3/2_ (853.8 eV) was well fitted with two peaks centered at 853.7eV and 854.6eV. Ni 2p_1/2_ was situated at 871.6 eV, accompanied by satellite peaks on the left side of the main peaks, which matched well with the corresponding values for the Ni^2+^ state previously reported [25]. The Fe 2p_3/2_ and Fe 2p_1/2_ spectra shown in Figure 2e present a pair of key peaks centered at 710.9 and 724.7 eV, except that a significant satellite peak related with typical Fe^3+^ at 718.5 eV [25,34]. It is more worthwhile to note that Ni 2p and Fe 2p spectra in NiFe_2_O_4_ nanocrystals show a positive shift of binding energy compared to NiFe, denoting higher valence states of Ni and Fe in NiFe_2_O_4_ nanocrystals [34]. Meanwhile, it also shows no chemical interaction with the surrounding Al_2_O_3_ matrix since the binding energy of the Al 2p spectrum is centered at 72.7 eV, and that of the O 1s spectrum is centered at 529.4 eV. Thus, the formation of NiFe/NiFe_2_O_4_ core/shell nanoparticles confined in the Al_2_O_3_ matrix was further demonstrated by XPS results.

**Figure 3:**
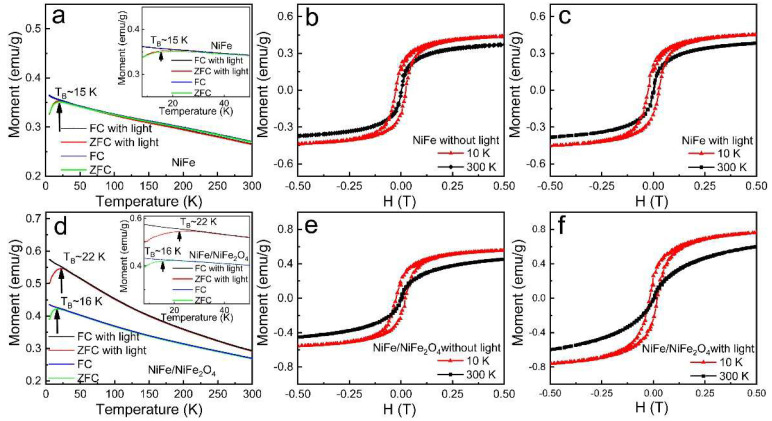
M–T curves (**a**,**d**), M–H curves (**b**,**c**,**e**,**f**) for NiFe nanoparticles and NiFe/NiFe_2_O_4_ core/shell nanoparticles with and without light, respectively. Inset of (**a**,**d**): corresponding magnified images of M-T curve at low-temperature range.

To characterize the ferromagnetic properties of NiFe nanoparticles and NiFe/NiFe_2_O_4_ core/shell nanoparticles, the measurement of field cooling (FC) and zero-field cooling (ZFC) modes from 5 K to 300 K were carried out, respectively. As shown in the ZFC curves in Figure 3a,d, both nanoparticles display a peak near the blocking temperature (T_B_). The ZFC curves are obviously separated from the FC curve below T_B_. Figure 3b,e show the magnetic-field dependent magnetization (M-H) measurements of NiFe nanoparticles and NiFe/NiFe_2_O_4_ core/shell nanoparticles at 10 K and 300 K, respectively. No significant hysteresis was observed for both samples at 300 K. However, at 10 K, obvious hysteresis can be seen for both samples with magnetic hysteresis loops, indicating the ferromagnetic behaviors. The M-T and M-H curves under light irradiation (530 nm, ~0.75 mW/cm^2^) were also shown in Figure 3d. Interestingly, under light irradiation, the M-T curve of NiFe/NiFe_2_O_4_ core/shell nanoparticles shows a much more obvious change compared with that of NiFe, where T_B_ is increased from ~16 K to ~22 K. Furthermore, it is worth noting that the saturation magnetization of NiFe/NiFe_2_O_4_ core/shell nanoparticles becomes larger under light irradiation. Meanwhile, M-T and M-H curves of NiFe nanoparticles show negligible change under light irradiation. Moreover, after turning off the light, the magnetization of both samples recovers rapidly to its no-light irradiation case. The above results clearly demonstrate that the magnetic change of NiFe/NiFe_2_O_4_ core/shell nanoparticles is purely electronic, unrelated to light or heat-induced structural or chemical changes [24]. Therefore, based on the above results, it can be inferred that the photogenerated electrons at the core/shell structure interface can improve ferromagnetism and T_B_.

**Figure 4:**
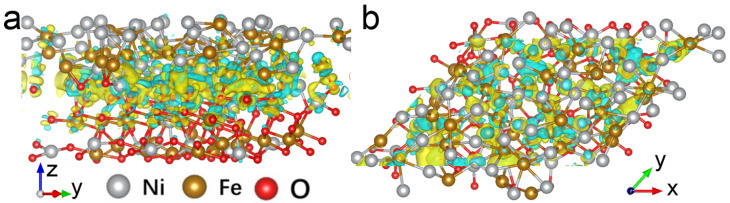
(**a**) Side and (**b**) top view of calculated charge density difference at the interface of NiFe and NiFe_2_O_4_. The blue and yellow regions represent electron depletion and accumulation, respectively.

In order to gain further insight into the enhanced ferromagnetism and T_B_ of NiFe/NiFe_2_O_4_ core/shell nanoparticles under light irradiation, the first-principles calculation was further carried out to investigate the electronic distribution at the interface of NiFe/NiFe_2_O_4_. The DFT calculation in this work was conducted using the Vienna ab initio simulation package (VASP) [35]. The interactions between core and electrons were described by employing the projector augmented wave (PAW) model with the Perdew–Burke–Ernzerhof (PBE) functional [36,37]. The plane wave expansion of the electronic wave function was set to an energy cutoff of 450 eV. Gamma-point centered Monkhorst-Pack grids of 2 × 3 × 1 k-point mesh were used to calculate slab geometry optimization. The number of atoms included in the calculations was 174. Lattice parameters were as follows: a = 17.33 Å, b = 12.92 Å and c = 27.72 Å. A vacuum layer of 15 Å thickness was added in the c direction. The force convergence criterion of 0.02 eV/Å of and the energy convergence criterion of 10^−5^ eV were set, respectively. The side and top views of calculated charge transfer and electron redistribution of NiFe/NiFe_2_O_4_ heterostructure are shown in Figure 4. It clearly shows that there is an obvious charge accumulation on the NiFe_2_O_4_ side at the interface of NiFe/NiFe_2_O_4_. Moreover, the density of states (DOS) of NiFe/NiFe_2_O_4_ core/shell nanoparticle (Figure S3 in Supplementary Materials) manifests the orbital hybridization effect on the interface, which may cause electron redistribution. Therefore, under light irradiation, much more photogenerated electrons are captured at the interface of NiFe/NiFe_2_O_4_, which improves the lower coordination of surface atoms, partially overcomes the surface spin disorder, and enhances ferromagnetism [24].

## 4. Conclusions

In summary, NiFe/NiFe_2_O_4_ core/shell nanoparticles were successfully synthesized by introducing oxygen during rapid thermal annealing. Obvious photo-modulated ferromagnetism was observed in core/shell nanoparticles confined in Al_2_O_3_ film. Given the interfacial effect, much more photogenerated electrons are captured at the interface, and the ferromagnetism of NiFe/NiFe_2_O_4_ core/shell nanoparticles can be improved by light irradiation. This work provides a promising strategy for the optical engineering design of optical information storage and magneto-optical coupling devices.

## Data Availability

Not applicable.

## Supplementary Materials

The following supporting information can be downloaded at: https://www.mdpi.com/article/10.3390/ma15041347/s1, Figure S1: Size distribution; Figure S2: Raman spectra; Figure S3: Calculated DOS of NiFe/NiFe_2_O_4_ core/shell nanoparticles.
