# Supplementary material for: Interfacial Effect on Photo-Modulated Magnetic Properties of Core/Shell-Structured NiFe/NiFe2O4 Nanoparticles
Source: Materials (Basel). 2022 Feb 11;15(4):1347. doi: 10.3390/ma15041347 (PMC8876216; doi:10.3390/ma15041347)
Supplement: Supplementary file 1 [file materials-15-01347-s001.zip › materials-1559193-supplementary.pdf]

materials

Supporting information

Interfacial effect on photo-modulated magnetic properties of core/shell-structured NiFe/$NiFe_2O_4$ nanoparticles

Wenda Zhou[1,2], Mingyue Chen[1,2], He Huang[1], Guyue Wang[1], Xingfang Luo[2], Cailei Yuan[2,*], Jingyan Zhang[1], Yanfei Wu[1], Xinqi Zheng[1], Jianxin Shen[1], Shouguo Wang[1,*], and Baogen Shen[3,1,4,*]

1 School of Materials Science and Engineering, Beijing Advanced Innovation Center for Materials Genome Engineering, University of Science and Technology Beijing, Beijing 100083, China
2 Jiangxi Key Laboratory of Nanomaterials and Sensors, School of Physics, Communication and Electronics, Jiangxi Normal University, 99 Ziyang Avenue, Nanchang 330022, Jiangxi, China
3 Beijing National Laboratory for Condensed Matter Physics, Institute of Physics, Chinese Academy of Sciences & University of Chinese Academy of Sciences, Beijing 100190, China
4 Institute of Rare Earths, Chinese Academy of Sciences, Jiangxi 341000, China
* Correspondence: clyuan@jxnu.edu.cn, sgwang@ustb.edu.cn, and shenbg@iphy.ac.cn

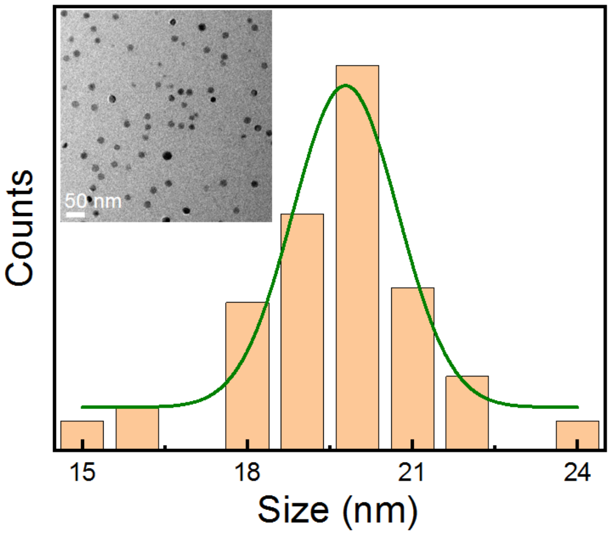

Figure S1 Size distribution of NiFe/$NiFe_2O_4$ core/shell nanoparticles and the corresponding zoom-in TEM image.

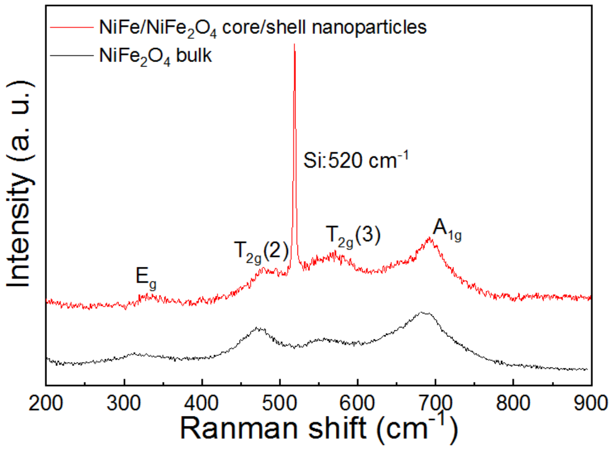

Figure S2 Raman spectra of $NiFe_2O_4$ bulk and $NiFe/NiFe_2O_4$ core/shell nanoparticles. A Si peak at 520 cm^{-1} is used as a reference for calibration.

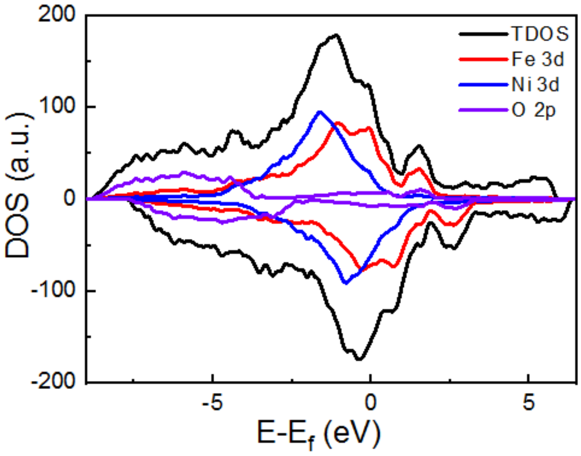

Figure S3 Calculated DOS of $NiFe/NiFe_2O_4$ core/shell nanoparticle.
